# Human IL-23R Cytokine-Binding Homology Region-Fc Fusion Protein Ameliorates Psoriasis via the Decrease of Systemic Th17 and ILC3 Cell Responses

**DOI:** 10.3390/ijms20174170

**Published:** 2019-08-26

**Authors:** Yue Gao, Zhengying Bian, Wenyao Xue, Qianwen Li, Yu Zeng, Yimeng Wang, Lei Tang, Tiejun Tang, Xiangdong Gao, Wei Guo

**Affiliations:** Jiangsu Key Laboratory of Druggability of Biopharmaceuticals and State Key Laboratory of Natural Medicines, School of Life Science and Technology, China Pharmaceutical University, Nanjing 210009, China

**Keywords:** psoriasis, IL-23/IL-17 axis, Th17 cells, adaptive immunity, innate immune

## Abstract

Interleukin (IL)-23 is considered an effective therapeutic target for the treatment of psoriasis because of the crucial role of the IL-23/IL-17 axis in the pathogenesis of psoriasis, and it has recently been reported to be involved in ILC3 cell differentiation. In this study, we report that eukaryotically expressed rhIL23R-CHR/Fc, as an endogenous extracellular receptor analogue, could be a natural antagonist in an imiquimod (IMQ)-induced psoriasis-like mouse model, including the antagonizing effect of suppressed inflammation in the skin lesion, decreased production of pro-inflammatory cells, and reduced the expression of pro-inflammatory factors. The rhIL23R-CHR/Fc fusion protein inhibits both innate immune and adaptive immune-mediated inflammatory responses. These findings shed light on rhIL23R-CHR/Fc as a promising candidate therapy for the treatment of psoriasis.

## 1. Introduction

Rigorous epidemiological studies have shown that autoimmune diseases affect more than 3–5% of the world’s population, and over 80 autoimmune diseases have been identified [1]. The causes of autoimmune diseases are extremely complicated, although genetic susceptibility and immunity and environmental risk factors have been linked to disease pathogenesis. Adaptive immune response, which is considered to be predominant in autoimmune disease [2], is involved in the formation of autoantibodies and antigen-specific T cell responses, resulting in the design of new drugs to suppress the function of T and B cells. However, clinical data indicated that these biological medicines could not achieve a complete cure. Emerging evidence suggests that innate immune activation also plays a pathogenic role in autoimmune diseases [3]. Accordingly, it is investigated whether we can design a biomacromolecule like the small molecule cyclosporin A (CsA) to correct the immune imbalance caused by both adaptive immunity and innate immunity. Simultaneously, due to the merits of definite, specific signal pathway inhibition, biopharmaceuticals can avoid the severe side effects caused by CsA.

Interleukin (IL)-23 is a heterodimeric cytokine composed of p19 subunit and p40 subunit shared by IL-12 and IL-23. It is well known that IL-23, mainly secreted by activated dendritic cells, macrophages, and keratinocytes in psoriatic skin lesions, contributes to the pathogenesis of various autoimmune diseases [4]. The genome-wide association study (GWAS) on psoriasis risk genetic loci also indicated that IL12B and IL23R are specifically susceptible genes associated with psoriasis [5]. Interleukin-23 binds IL-23 receptor (IL-23R) to activate the JAK2/STAT3 pathway, which is essential for the survival and expansion of IL-17-producing T helper (Th) 17 cells [6]. Meanwhile, IL-23 is regarded as the most potent cytokine of IL-17-producing cells [7], such as Th17 cells and cytotoxic T (Tc) 17 cells. Therefore, a number of biopharmaceuticals, such as guselkumab, tildrakizumab, and risankizumab [8,9,10], targeting IL-23p19 have shown considerable clinical efficacy for autoimmune diseases. However, psoriasis cannot be completely cured because of the deletion of existing therapeutic drugs and lack of diagnostic markers for psoriasis.

Recently, it has been reported that IL-23 can be a master mediator for the activation of innate lymphoid cells (ILCs) [11]. These are a newly discovered class of lymphocyte subsets that originate from common lymphoid progenitor cells [12]. As innate immune cells, ILCs play an important role in the early stage of the immune response and are the main sources of various pro-inflammatory and anti-inflammatory cytokines [13]. Similar to Th17 cells, based on the master transcription factor RAR-related orphan receptor gamma t (RORγt) [14,15], ILC3s, a subset of ILCs, exhibit remarkably similar transcriptional profiles and secrete IL-17/IL-22 to activate effector functions in a STAT3-dependent manner in response to IL-23. Meanwhile, innate lymphoid cells regulate adaptive lymphocyte responses through the production of cytokine, expression of co-stimulatory/inhibitory molecules, and antigen presentation [16]. Coordinating the interactions between ILCs and T cells could promote host protection and prevent autoimmune diseases [17].

There is clear clinical evidence that biological agents from the market that specifically block IL-23p19 subunits are effective and superior to other downstream cytokines acting on the IL-23/IL-17 axis, but there are few data to show its analogous effect on innate immunity. We previously constructed and expressed a IL-23 soluble receptor molecule described as human interleukin 23-receptor cytokine receptor-binding homology region (hIL-23R-CHR) [18], which can directly antagonize IL-23, significantly improve the symptoms of various autoimmune diseases in model mice, and decrease the incidence through the IL-23/IL-17 pathway [19,20]. In this study, we further investigated whether the eukaryotically expressed rhIL-23R-CHR/Fc could be a natural antagonist in an imiquimod (IMQ)-induced psoriasis-like mouse model, more specifically whether rhIL-23R-CHR/Fc could affect the activation of ILC3 cells while interfering with Th17 subsets.

To investigate the connection between the abnormal innate immune and adaptive immune activation in psoriasis, in particular to explore the mechanism for Th17 and ILC3 dysregulation in psoriasis, we established an IMQ-induced psoriasis-like mouse model. The rhIL-23R-CHR/Fc significantly improved psoriasis lesions caused by abnormal immune activation. Additionally, our data indicated that rhIL-23R-CHR/Fc suppresses Th17 cell differentiation and reduces the transcriptional and protein levels of pro-inflammatory cytokines. Moreover, blockage of IL-23 could decrease ILC3s in the lamina propria of the small intestine, particularly suppressed mIL-22-secreting natural cytotoxic receptor (Nkp46)-ILC3 cells. Our present study has highlighted the possibility of using rhIL-23R-CHR/Fc to treat psoriasis, and further demonstrated that rhIL-23R-CHR/Fc could improve IMQ-induced psoriasis through not only the inhibition of the adaptive immunity of the IL-23/Th17 pathway but also the inhibition of the innate immunity of ILC3 activation, which provides a new direction for drug design for autoimmune diseases.

## 2. Results

### 2.1. Construction, Expression and Purification of rhIL-23R-CHR/Fc

**Figure 1:**
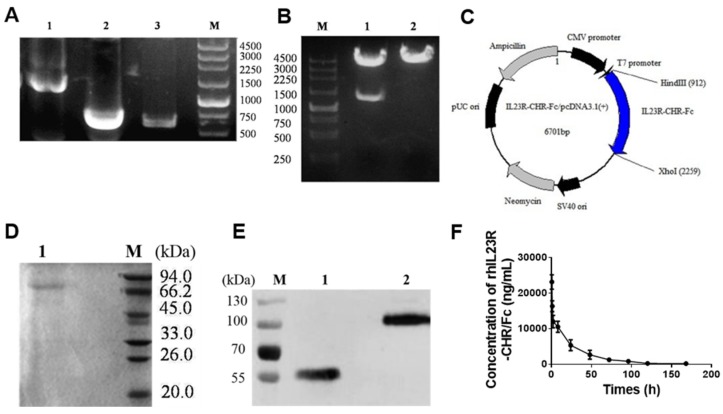
Construction, expression, and purification of rhIL-23R-CHR/Fc. (**A**) PCR products of rhIL-23R-CHR/Fc recombinant gene. Lane 1, rhIL-23R-CHR/Fc gene sequence. Lane 2, hIgG1 Fc fragment. Lane 3, rhIL-23R-CHR gene. M, DNA molecular weight markers, bp. (**B**) Identification of rhIL-23R-CHR-Fc/T vector digested by Hind III and Xho I sites. Lane 1, pcDNA3.1 (+) - IL-23R-CHR/Fc after digestion. Lane 2, pcDNA3.1 (+) - IL-23R-CHR/Fc before digestion. M, DNA marker, bp. (**C**) Plasmid map of pcDNA3.1 (+) - IL-23R-CHR/Fc. The gene sequence encoding rhIL-23R-CHR/Fc was inserted into pcDNA3.1 (+) vector at the corresponding restriction sites Hind III and Xho I. (**D**) SDS-PAGE analysis of the purified rhIL-23R-CHR/Fc fusion protein using Protein A column. Lane 1, purified rhIL-23R-CHR/Fc fusion protein at reduced state. M, protein molecular weight markers, KDa. (**E**) Western blot analysis of rhIL-23R-CHR/Fc using mAbs against human IL-23R. Lane 1, purified rhIL-23R-CHR/Fc fusion protein at non-reduced state. Lane 2, purified rhIL-23R-CHR/Fc fusion protein at reduced state. (**F**) Plasma clearance of IL23RCHR-Fc in mice.

Based on previous construction and expression of hIL-23R-CHR in *Escherichia coli*, we designed a rhIL-23-CHR/Fc protein containing hIL-23R-CHR and fused it with an Fc fragment for stronger stability and easy preparation in CHO cells. The genes of rhIL-23R-CHR (including the signal peptide, 648 bp and hIgG1 Fc, 699 bp) were obtained by PCR as previously described. After overlap PCR was used to construct rhIL-23R-CHR/Fc (1347 bp), agarose electrophoresis results showed a band of the expected size (Figure 1A). Then, the gene was cloned into pcDNA3.1 (+) at the restriction digestion sites Xho I and Hind III, and the gene sequence was confirmed by restriction digestion (Figure 1B) and DNA sequencing. The map of rhIL-23R-CHRFc/pcDNA3.1 (+) plasmid is shown in Figure 1C. Next, a stable expression cell line was established after screening against 400 μg/mL G418 sulfate, and the supernatants of cell cultures were purified by Protein-A affinity chromatography. 

Finally, recombinant protein was obtained with a purity of approximately 90% on SDS-PAGE, and its identity was verified by Western blot (Figure 1D,E). The molecular weight under the non-reduced condition was approximately twice that of the reduced conditions (Figure 1E), which may form a dimer through a disulfide linkage between the cysteine residues at the N-terminus of Fc fragment. This dimeric feature may possess advantages of stability and in vivo behaviors. It is worth noting that the actual molecular weight (60 kDa) on Western blot was larger than its theoretical molecular weight (48.17 kDa), most likely due to the glycosylation modification of the fusion protein. In addition, our results showed that the half-life of rhIL-23R-CHR/Fc in vivo is about 72 h (Figure 1F). The half-life of the rhIL-23R-CHR/Fc was significantly longer compared to the IL-23R-CHR protein by prokaryotic expression. Then, we tried to verify whether the fusion protein exhibited the desired biological activity and function. 

### 2.2. Inhibition of Th17 Cell Differentiation by rhIL-23R-CHR/Fc Fusion Protein

**Figure 2:**
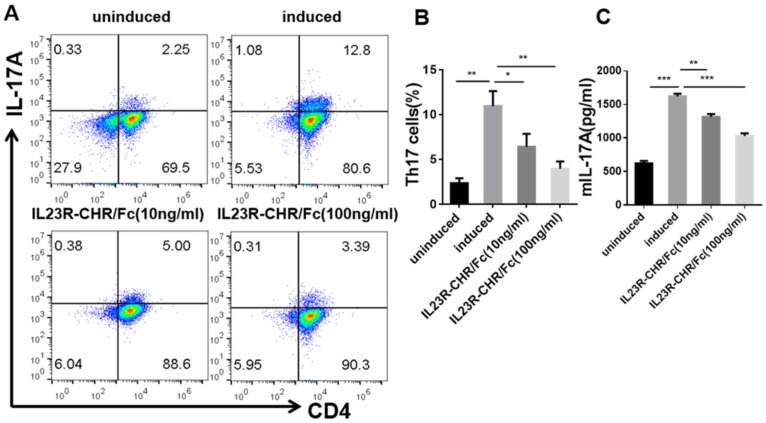
Role of rhIL-23R-CHR/Fc in mouse Th17 cell differentiation. Mouse naive CD4^+^ T cells were differentiated into Th17 cells in the presence of different concentrations of rhIL23R-CHR/Fc in vitro. (**A**) Analysis of Th17 cells (CD4^+^ IL-17^+^) by flow cytometry. (**B**) The percentage of Th17 cells. (**C**) IL-17A level in supernatants derived from cultured spleen cells. All tests were performed in triplicate and are presented as the mean ± SEM. * *p* < 0.05; ** *p* < 0.01; and *** *p* < 0.001.

To determine whether the blockade effects of IL-23 were associated with Th17 cell differentiation, purified mouse naive CD4^+^ T cells were cultured in the presence or absence of the rhIL-23R-CHR/Fc protein under Th17-polarizing conditions. As expected, with the increase of recombinant protein concentration, rhIL-23R-CHR/Fc fusion protein suppressed mouse Th17 cell differentiation in a dose-dependent manner (Figure 2A,B). With the treatment of rhIL-23R-CHR/Fc, IL-17A secretion from the cells also remarkably decreased, as shown in Figure 2C. When the concentration of rhIL-23R-CHR/Fc was increased from 10 ng/mL to 100 ng/mL, the inhibition rate of mIL-17 secretion increased from 14.4% to 32.7% (Figure 2C). These data collectively demonstrated that rhIL-23R-CHR/Fc could inhibit mouse Th17 cell differentiation in a dose-dependent manner.

### 2.3. Amelioration of Skin Lesions in a Mouse Psoriasis Model by rhIL-23R-CHR/Fc

**Figure 3:**
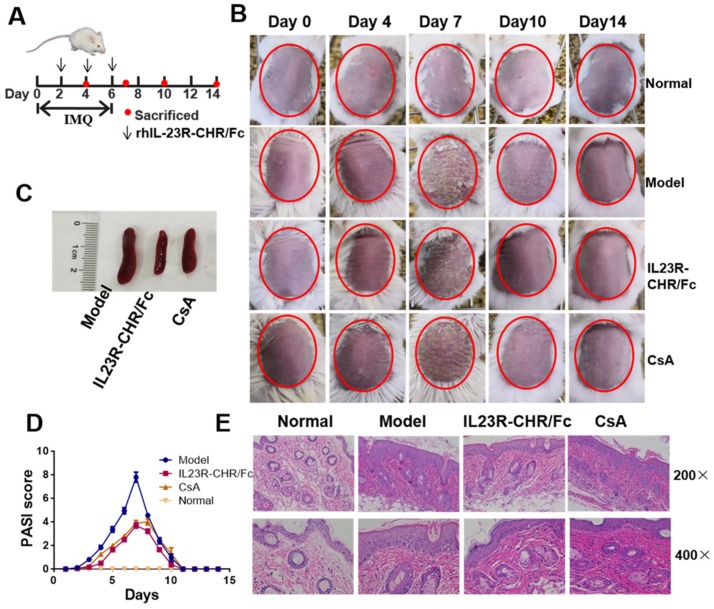
rhIL-23R-CHR/Fc ameliorated skin inflammation in an imiquimod (IMQ)-induced psoriasis-like model. (**A**) Schematic diagram of intravenous administration of rhIL23R-CHR/Fc on days 2, 4 and 6 during the application of IMQ. Three mice in each group were sacrificed on days 4, 7, 10 and 14 to conduct experiments. The normal group did not receive IMQ as a negative control. Cyclosporin A (CsA), an immunosuppressive agent commonly used in the clinical therapy for autoimmune diseases, was used in the treatment of psoriasis at 1.5 mg/kg as a positive control. (**B**) Phenotypic presentation of lesional skin from mice. (**C**) The size of spleens of mice. (**D**) Mice skin Psoriasis Area and Severity Index (PASI) scores. (**E**) Haematoxylin and eosin staining of lesional skin obtained from normal mice or mice treated with IMQ, CsA or rhIL-23R-CHR/Fc at day 7 (200×, 400×).

To investigate the inhibitory effects of rhIL-23R-CHR/Fc on psoriasis development in animals, we chose an IMQ-induced psoriasis-like mouse model (Figure 3A), which closely resembles the human disease phenotype according to a previous report [21]. The application of IMQ on mouse back skin led to significant thickening, redness, and scaling (Figure 3B). To evaluate the efficacy of rhIL-23R-CHR/Fc in the IMQ-induced psoriasis-like model, we intravenously injected rhIL-23R-CHR/Fc into model mice, and the mice were sacrificed at 4, 7, 10 and 14 days (Figure 3A). As anticipated, the rhIL-23R-CHR/Fc-treated group did not develop typical psoriasis-like skin lesions compared to the model group (Figure 3B). On day 7, the peak of psoriasis-like changes, the splenomegaly (Figure 3C) of mice in the rhIL-23R-CHR/Fc-treated group was significantly ameliorated compared to the model group. Based on clinical Psoriasis Area and Severity Index (PASI) for psoriasis patients, we used an objective scoring system [22] to compare the severity of back skin inflammation between treatment and model. The administration of rhIL-23R-CHR/Fc also delayed psoriasis-like pathological progression and reduced disease severity (Figure 3D). 

Histological analysis of lesion skin using haematoxylin and eosin staining showed a decrease of inflammatory cell infiltration in rhIL-23R-CHR/Fc-treated mice compared to the model group on day 7 (Figure 3E). In addition, the characteristic epidermal cell hyperplasia induced by IMQ was alleviated after rhIL-23R-CHR/Fc treatment (Figure 3E). Taken together, these results indicate that the present rhIL-23R-CHR/Fc can prevent the immune pathological changes in psoriasis and effectively ameliorate plaque formation. 

### 2.4. Suppression of the Proportion of IL-17-Producing Cells by rhIL-23R-CHR/Fc

**Figure 4:**
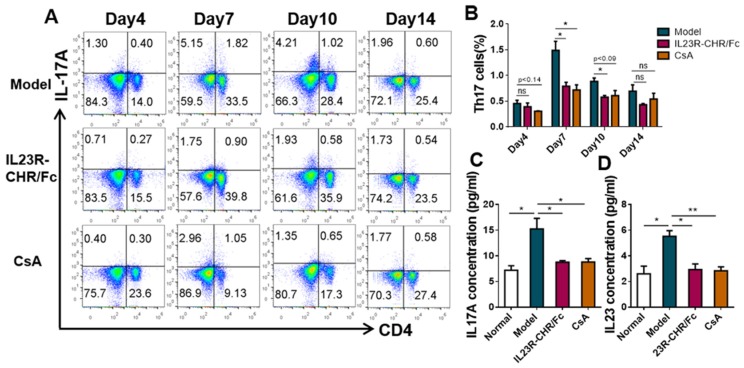
rhIL-23R-CHR/Fc inhibited Th17 cell-mediated inflammatory response. (**A**) Representative flow-cytometric analysis of Th17 cells (CD4^+^IL-17A^+^) in spleen from normal mice or mice (*n* = 3) treated with IMQ at day 4, 7, 10, and 14. (**B**) Statistical data of Th17 cell percentage. (**C**,**D**) Protein levels of mIL-17A(C) and mIL-23(D) in mice serum. Data are mean ± SEM of *n* ≥ 3 per group. * *p*< 0.05, ** *p* < 0.01, and *** *p* < 0.001. ns, not significant (Student’s *t*-test).

**Figure 5:**
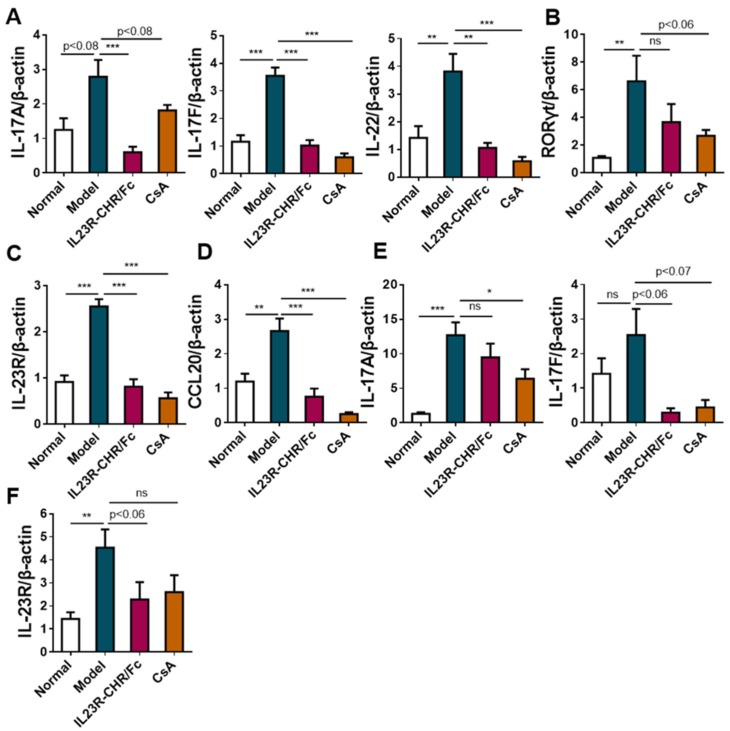
rhIL-23R-CHR/Fc inhibited the transcriptional levels of inflammatory-associated cytokines, transcription factors, and chemokines in mouse skin and spleen. (**A**–**D**) The mRNA expression of IL-17A, IL-17F, IL-22 (**A**), Rorγt (**B**), IL-23R (**C**) and CCL20 (**D**) in skin lesions from normal or mice treated with IMQ (*n* > 3). (**E**,**F**) The mRNA expression of IL-17A, IL-17F (**E**), and IL-23R (**F**) in spleen from normal or mice treated with IMQ. All results are representative of at least 3 independent experiments with at least 3 samples in each group. Data are mean ± SEM. * *p* < 0.05, ** *p* < 0.01 and *** *p* < 0.001. ns, not significant (Student’s *t*-test).

Interleukin (IL)-23 is a key regulator of Th17 cell differentiation and development. Over the course of the animal experiments, the percentage of Th17 cells were much lower on day 4 to day 14 in the rhIL-23R-CHR/Fc-treated group than that in the model group (Figure 4A,B), along with decreased expression of IL-17 (Figure 4C) and IL-23 (Figure 4D) in mouse serum. Results suggested that Th17 cells may initiate and aggravate the progression of psoriasis. To confirm the effects of rhIL-23R-CHR/Fc on inflammatory infiltration in psoriasis skin, we took mouse skin and analyzed the transcription level of Th17 cells-related genes. As a result, mRNA levels of cytokines IL-17A, IL-17F, IL-22 (Figure 5A), transcription factor RORγt (Figure 5B), cytokines receptor IL-23R (Figure 5C), and chemokine CCL20 (Figure 5D) in skin was significantly lower, accompanied by decreased IL-17A, IL-17F, and IL-23R mRNA expression, in spleen of the mice treated with rhIL-23R-CHR/Fc (Figure 5E,F).

**Figure 6:**
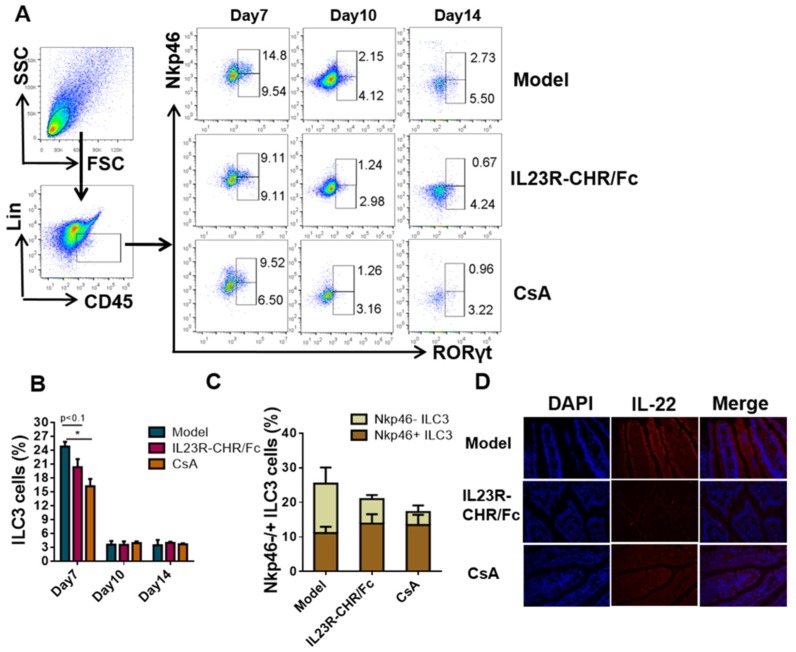
rhIL-23R-CHR/Fc limited the production of ILC3 cells. (**A**) Percentages of ILC3s (Lin^−^ CD45^+^ RORγt^+^) in small intestine lamina propria from Normal, Model, rhIL-23R-CHR/Fc, and CsA group mice were analyzed by flow cytometry (*n* = 3) at day 7, 10, and 14. (**B**) Statistical data of ILC3 cells percentage. (**C**) The frequency of Nkp46^−^/^+^ILC3 cells at day 7 from Normal, Model, rhIL-23R-CHR/Fc, and CsA group mice. (**D**) Analysis of IL-22 level in small intestines by immunofluorescence staining (200×). All results are representative of at least 3 independent experiments with at least 3 samples per group in each. Data represent the mean ± SEM. * *p* < 0.05 (Student’s *t*-test).

To further examine the potential influence on the innate counterparts of Th17 cells-ILC3 cells by rhIL-23R-CHR/Fc, we isolated and counted lamina propria lymphocytes from small intestines. Interestingly, antagonism of IL-23 by rhIL-23R-CHR/Fc remarkably decreased the number of ILC3 cells (Figure 6A,B). More importantly, rhIL-23R-CHR/Fc could reduce the proportion of Nkp46^−^ ILC3 cells in comparison with the model mice (Figure 6C). The effector function of ILC3s are largely defined by the cytokines they produce. Because IL-22 has a detrimental role in psoriasis [23], the IL-22 level in small intestines was significantly inhibited in rhIL-23R-CHR/Fc and CsA-treated mice compared to that in the model group according to immunofluorescence analysis of the small intestine (Figure 6D). Together, these findings strongly confirm that the systematic aberrant activation and differentiation of CD4^+^ T cells, particularly IL-17-producing cells, play a master role in the formation of psoriasis lesions, and the blockade of IL-23 cytokine can significantly delay the development of psoriasis induced by IMQ. 

## 3. Discussion

Because psoriasis is one of the most common autoimmune diseases, a broader and deeper understanding of the immunological mechanisms leading to plaque formation requires further and detailed elucidation. Current treatment of psoriasis is primarily based on the severity of the disease. Local and systemic treatments as well as phototherapy are used for the treatment of psoriasis. Methotrexate, cyclosporine, acitretin, and etretinate are commonly used first-line drugs [24]. With the exploration of Th17 cells, the research process of autoimmune diseases has been greatly promoted. Recently developed biologics targeting the IL-23/IL-17 axis offer new and diverse therapeutic options for patients with autoimmune diseases such as psoriasis and rheumatoid arthritis [25]. Consequently, proliferation-inducing mediators such as IL-17, tumor necrosis factor (TNF)-α, IL-23, and related receptors expressed by immune cells have become therapeutic targets for newly approved anti-psoriatic therapies. 

Apart from adaptive immunity, innate immune cells also play a crucial role in the pathogenesis of psoriasis. Current research showed that the accumulation and activation of ILC3 cells in experimental autoimmune encephalomyelitis (EAE) [26], circulating ILC3s, as a major source of IL-17/IL-22, have recently been identified as associating with psoriasis and psoriatic arthritis [27,28]. Because activated ILC3 cells secrete cytokines IL-17 or IL-22 in response to IL-23 and/or IL-1β, our present study intended to determine whether rhIL-23R-CHR/Fc could interfere with the activation of both ILC3 cells and Th17 cells via the IL-23/IL-17 axis. Flow-cytometry results showed that rhIL-23R-CHR/Fc suppressed not only the classic immune pathway of the IL-23/Th17 pathway but also the innate immune mediated by ILC3 (Figure 4A and Figure 6A). These ILC3s are mainly distributed in the lamina propria of the small intestine [29] and are responsible for intestinal mucosal immunity. The available evidence indicated that IMQ-induced disease mice exhibit accelerated DSS colitis, in which skin inflammation may contribute to pathogenic conditions in the gut [30], suggesting a potential skin-intestinal interaction during the pathogenesis of psoriasis. Our study similarly found that severe intestinal inflammation and a significant increase in the frequency of ILC3s were observed in the psoriasis-like mice (Figure 6B), which suggested interaction between skin and intestine. 

According to the differential expression of chemokine receptor 6 (CCR6) and natural cytotoxic receptor (NCR), ILC3s are divided into three different cell subtypes, including lymphoid-tissue inducer (LTi), NCR^−^ ILC3s, and NCR^+^ ILC3s. Statistical analysis of clinical data have indicated a significant increase of circulating NCR^+^ILC3 in the blood of psoriasis patients compared to healthy individuals, suggesting a crucial role for NCR^+^ ILC3 in psoriasis pathogenesis [31,32]. Strikingly, ILC3s differ in mice and humans. The IL-22 expression is confined to NKp44^+^ ILC3 cell subsets in humans [33]. However, IL-22 is preferentially expressed by NKp46^−^ ILC3s in mice [34]. Flow cytometry and immunofluorescence results showed that rhIL-23R-CHR/Fc strictly decreased IL-22-producing Nkp46^−^ ILC3 cell subsets, providing more evidence for species difference (Figure 6C). Based on the above results, we propose the mechanism of rhIL-23R-CHR/Fc in the treatment of psoriasis: On the one hand, rhIL-23R-CHR/Fc binds to free IL-23 to directly inhibit the differentiation of Th17 cells; on the other hand, rhIL-23R-CHR/Fc suppresses the activation of IL-22-secreting ILC3 cells and indirectly affects the pathogenesis of the disease through skin-intestinal interaction. In conclusion, rhIL-23R-CHR/Fc regulates adaptive and innate immune responses by reducing the frequency of Th17 and ILC3 cells, and then inhibiting their function to secrete pro-inflammatory cytokines. Nevertheless, whether there is reciprocal interaction between ILC3 cells and Th17 cells in preventing autoimmune diseases requires further study. Moreover, whether activated ILC3s further regulate adaptive lymphocyte responses through the production of cytokine, antigen presentation, and expression of co-stimulatory/inhibitory molecules needs to be examined.

The available results indicate that the expression of IL-23 is increased in model mice skin lesions, whereas dermatitis is almost completely blocked in IL-23p19 receptor-deficient mice [35]. Similarly, our present results found that both rhIL23R-CHR/Fc and CsA treatments prevented disease progression and ameliorated disease severity (Figure 3B). Given that IL-23 participated in the differentiation and activation of both Th17 and ILC3 cells and promoted the abnormal expansion of keratinocytes through subsequently up-regulating the secretion of IL-17 and IL-22, we paid special attention to the intervention effect of inflammatory factors acting at different levels from mRNA to protein. Further, rhIL23R-CHR/Fc significantly inhibited the mRNA level of transcription factors, chemokine, and cytokines represented by RORγt, CCL20, and IL-17 (Figure 5A–F) and suppressed the expression of circulating IL-17 and IL-23 (Figure 4C,D). Our present study has demonstrated the crucial roles of Th17 cells and ILC3 cells in the development of psoriasis skin lesion, which should be helpful to better understand the pathological process and therapeutic intervention. The homolog of IL-23R-CHR genes between human and mouse is 84%, this human recombinant protein sequence could serve as a molecular probe to investigate the therapeutic effect of IL-23 antagonist in autoimmune diseases characterized by high expression of IL-23 and abnormal proliferation of Th17 cells.

In conclusion, we have constructed a rhIL-23R-CHR/Fc fusion protein to specifically bind IL-23 in a eukaryotic expression system. The fusion protein exhibited significant inhibitory effects on Th17 cell differentiation and IL-17 production in vitro. In addition, rhIL-23R-CHR/Fc almost completely ameliorated the development of psoriasis and improved psoriasis-associated histological changes via inhibiting Th17 and ILC3-mediated pro-inflammatory responses, and the inhibition rates were 51% and 28%, respectively. The Th17 and ILC3 cells play an interactive role in the pathogenesis of psoriasis, whereas fusion proteins mainly suppress adaptive immunity responses. Collectively, the present results strongly suggest that rhIL-23R-CHR/Fc could inhibit or reverse the pro-inflammatory responses mediated by adaptive and innate immunity in psoriasis and could be a promising therapy for autoimmune diseases, which may offer diverse benefits compared to typical immunosuppressant drugs.

## 4. Materials and Methods

### 4.1. Construction, Expression and Purification of rhIL-23R-CHR/Fc

Our previous study described the construction and expression of rhIL-23R-CHR in prokaryotic [18]. Using the plasmid template of rhIL-23R-CHR gene containing the signal peptide (GenBank: NP_653302.2) and the hIgG-Fc (GenBank: AEV43323.1) gene, we obtained a fully humanized IL-23R-CHR/Fc fusion gene by twice overlap polymerase chain reaction (PCR) extension using the primers as follows (Table 1). The rhIL23R-CHR/Fc fragment cleaved from rhIL-23R-CHR/Fc/T Vector pMD19 (Takara, Tokyo, Japan) with Xho I and Hind III (Takara, Tokyo, Japan). Correct rhIL-23R-CHR/Fc gene fragment was confirmed by sequencing and then inserted into pcDNA3.1 (+) (Invitrogen, Carlsbad, CA, USA) eukaryotic expression vector *Escherichia coli*. The recombinant plasmid transformed into DH5α (Novagen, Darmstadt, Germany) and the positive selection was conducted on Luria-Bertani culture supplemented with 0.1 mg/mL ampicillin (Sigma, St. Louis, MO, USA). 

The endofree and linearized plasmids prepared by Pvu I (Takara, Tokyo, Japan) were transfected into the CHO-k cell (ATCC, Manassas, VA, USA) at the logarithmic growth phase using PEI (Polysciences, Chicago, IL, USA). After 24 h, the medium contained 400 μg/mL G418 sulfate (Gibco, Grand Island, NY, USA) was used to select monoclonal cell lines. CHO-k cells which stably expressed the interest protein were identified by Western blot.

Later, the medium was changed to the SFM medium (Gibco, USA) without FBS for additional 48 h. Cells culture was harvested by centrifugation at 8000 rpm for 15 min. Operation of protein A Sepharose column has been described in our previous study [36]. In brief, the Protein A Sepharose column (GE Healthcare, Piscataway, NJ, USA) was pre-equilibrated with binding buffer (20 mmol/L NaH_2_PO_4_, 20 mmol/L Na_2_HPO_4_, pH 7.0) at a flow rate of 1 mL/min. The resultant supernatant was loaded onto the column after filtering through a 0.45 μm filter, Proteins specifically bound to the resin were eluted with 0.1 mol/L Glycine, pH 2.8, and immediately neutralized with 1 mol/L Tris HCl pH 9.0.

### 4.2. Differentiation of Th17 Cell in Vitro

C57BL/6 mice (Yangzhou University Comparative Medical Research Center, Yangzhou, China) spleens were teased through sterilized 70 μm cell strainers (BD Biosciences, San Jose, CA, USA) to obtain single-cell suspensions in IMDM medium (Gibco, Grand Island, NY, USA) containing 10% FBS (Gibco, Grand Island, NY, USA). RBC lysis buffer (BD Pharmingen, San Diego, CA, USA) lysed red blood cells. Anti-CD4 magnetic beads (Miltenyi biotech, Bergisch Gladbach, Germany) was used to purify CD4^+^ T cells. Mixed lymphocyte were stained with APC-adjusted anti-mouse CD4 antibody (BD Biosciences, San Jose, CA, USA) to detect the purity of CD4^+^ T cells. For mouse Th17 cell differentiation, naïve CD4^+^T cells were seeded in 24 well plates at 10^6^/well and stimulated with plate-bound 1 μg/mL anti-mCD3 and 1 μg/mL soluble anti-mCD28 (eBioscience, San Diego, CA, USA) under Th17 polarizing conditions: 1 ng/mL mTGF-β, 10 ng/mL mIL-6, and 10 ng/mL mIL-23 (R&D, Minneapolis, MN, USA) for 72 h. Simultaneous treatment with different concentrations of rhIL-23R-CHR/Fc protein. The endotoxin was removed for all proteins with Endotoxin affinity Resin (Genscript, Piscataway, NJ, USA) before assays.

### 4.3. Western Blot

Cells treated with or without rhIL-23R-CHR/Fc were collected and lysed with radio immunoprecipitation assay lysis buffer supplemented with 1 mmol/L PMSF (Beyotime, Shanghai, China) for 30 min in ice. The lysate was centrifuged at 12,000 rpm for 10 min at 4 °C, and the supernatant was removed. Proteins were quantified by the BCA (Thermo, Waltham, MA, USA). The total protein was separated via electrophoresis on 10% SDS-PAGE, then electro blotted onto PVDF membranes (Merck Millipore, Billerica, MA, USA) and probed using goat anti-IL-23R Ab (Santa Cruz Biotechnology, Santa Cruz, CA USA). Quantification of IL23R was normalized to β-actin (ZSGB, Beijing, China) by grayscale.

### 4.4. Pharmacokinetic Studies

A preliminary pharmacokinetic study was performed using 6- to 8-week-old ICR mice (Yangzhou University Comparative Medical Research Center, Yangzhou, China) that were injected with rhIL-23R-CHR/Fc at 1 mg/kg via tail vein. Then blood samples were collected at 0.5, 1, 2, 8, 24, 48, 72, 96 and 120 h after injection and centrifuged at 6000 rpm for 10 min to obtain serum. The serum concentrations of rhIL-23R-CHR/Fc were measured using ELISA kits (Dakewe, Shenzhen, China) under the manufacturers’ instructions. Pharmacokinetic parameters were calculated from Winnonlin 5.2 (Mountain View, CA, USA) and dose concentration curves were plotted as GraphPad Prism 6.0 (San Diego, CA, USA).

### 4.5. Induction of Psoriasis-Like Mouse Model and Treatments

Six to eight-week-old female BALB/c mice were purchased from Vital River (Beijing, China), were used for establishing the IMQ-induced psoriasis-like model. All mice were maintained in specific pathogen-free conditions. All animal procedures meet the Guidelines for the Care and Use of Laboratory Animals as adopted and promulgated by the United States National Institutes of Health, and approved by the Jiangsu Provincial Experimental Animal Manage Committee under the contract of SCXK (su) 2016-0003.

Mice were kept under controlled conditions. The model mice were treated with a daily topical dose of 5% IMQ cream (62.5 mg/d) (Sichuan Med-shine Pharmaceutical, H20030128, China) on the shaved back of mice for 6 consecutive days. Normal group was treated with the same dose of vehicle cream. For the treatment of psoriasis, rhIL23R-CHR/Fc was administered via tail vein injection at 2.5 mg/kg on days 2, 4 and 6 during the application of IMQ. Then, 1.5 mg/kg CsA (Shanghai yuanye, Shanghai, China) was administrated by intragastric once a day during the application of IMQ as positive control. Mice were given normal diet and water during the experiment. At least three mice in each group were sacrificed for tissue and serum harvest on days 4, 7, 10 and 14. Mice erythema, scaling, and thickening were scored respectively on a scale from 0 to 4:0, none; 1, slight; 2, moderate; 3, marked; 4, very marked in accordance with the clinical PASI for psoriasis patients. The total score was calculated by adding the 3 index scores together (score 0-12).

### 4.6. Isolation of Murine Small Intestinal Lamina Propria Lymphocyte

Isolation of lamina propria cells was performed as described [37]. After removing the intestines contents and fat, inverted intestines were extracted with 5% (w/v) dithiothreitol (DTT) (DINGGUO, Beijing, China) and 0.5 M EDTA (Nanjing Huaxueshiji, Nanjing, China) in RPMI (Bioind, Kibbutz Beit Haemek, Israel) supplemented with 10% FBS for approximately 15 min at 37 °C under gentle agitation. Then tissue debris were digested with collagenase II (1.5 mg/mL; Sigma, St. Louis, MO, USA) and dispase (0.5 mg/mL; Sigma, St. Louis, MO, USA) in RPMI supplemented with 10% FBS for approximately 30 min at 37 °C under gentle agitation. Filter digested tissue through a 40 μm cell strainer. The strainer was rinsed with RPMI containing 10% FBS and the filtered solution centrifuged at 500 g for 10 min at 4 °C. Pellet was resuspended in 1 mL of RPMI complete medium.

### 4.7. Flow Cytometry

Spleen cells and small intestinal lamina propria cells were washed with PBS buffer and centrifuged at 400 g for 5 min. Cytokines, transcription factors, and surface markers were evaluated using Accuri C6 flow cytometer (BD Biosciences, San Jose, CA, USA). For cytokine detection, cells were stimulated for 5 h with PMA and ionomycin with the addition of GolgiPlug (BD Biosciences, San Jose, CA, USA) and Monensin Sodium (MultiSciences, Hangzhou, China). Then, cells were incubated with surface markers’ antibodies for 30 min. Cells were fixed and permeabilized with a mouse Cytofix/Cytoperm (BD Biosciences, San Jose, CA, USA) for intracellular staining or Transcription Factor Buffer Set (BD Biosciences, San Jose, CA, USA) for transcription factors staining and then stained with fluorescent antibodies against cytokines or transcription factors for an additional 30 min. All antibodies staining procedures were incubated on ice in the dark. Events were analyzed by FlowJo software (TreeStar, Ashland, OR, USA). Lineage cocktail antibody contains anti-CD3e, anti-CD11b, anti-CD45R/B220, anti-Ly-76, and anti- Ly-6G and Ly-6C.The following antibodies were used: APC anti-mouse CD4 (catalog 553051), PE anti-mouse IL-17A (catalog 559502), Alexa Fluor 647 anti-mouse CD335 (catalog 560755), PE anti-mouse RORγt (catalog 562607), FITC anti-mouse CD45 (catalog 553079), and PerCP-Cy5.5 anti-mouse Lin (catalog 561317) were purchased from BD Biosciences (San Jose, CA, USA).

### 4.8. Quantitative Real-Time Polymerase Chain Reaction (qPCR)

Total RNA was extracted from spleen or skin samples using EasyPure RNA Kit (TransGenBiotech, Beijing, China) and a NanoDrop spectrophotometer (ND-2000, Thermo Fisher Scientific, Waltham, MA, USA) was used for RNA quality control. RNA was reverse-transcribed with the HiScript II Q RT SuperMix for qPCR (Vazyme, Nanjing, China) and each test consumed 1 μg of total RNA according to the manufacturer’s instructions. The quantitative RT-PCR was carried out on an Applied Biosystems QuantStudio 3 Real-Time PCR Instrument (Thermo Fisher Scientific, Waltham, MA, USA) with the 2×ChamQTM Universal SYBR^®^ qPCR Master Mix (Vazyme, Nanjing, China). Each amplified sample was performed in triplicate and the relative expression level of gene was determined by normalizing to β-actin expression measured contemporaneously from the same sample. The fold change of gene expression was calculated by 2^-(ΔCt experimental group-ΔCt normal group), which normalized to the normal group. Primers for interest gene were synthesised from GenScript (Piscataway, NJ, USA). Sequences of PCR primer pairs were as shown in Table 2.

### 4.9. ELISA

The level of mIL-17A in cell supernatant was measured using mouse IL-17A pre-coated ELISA kit (Dakewe, Shenzhen, China). The levels of mIL-17A and mIL-23 in mice serum was measured using sandwich ELISA assays with commercially available ELISA kits (MultiSciences, Hangzhou, China) according to the manufacturers’ instructions.

### 4.10. Histology Analysis

Mouse skins were fixed in 4% PFA (Servicebio, Wuhan, China) for 24 h andembedded in paraffin. Then skins in paraffin were sectioned and stained with hematoxylin and eosin (Servicebio, Wuhan, China) according to standard laboratory procedures.

### 4.11. Immunofluorescence Analysis

Tissues were isolated and fixed with 4% PFA for 24 h at room temperature, washed twice with PBS and stored using 75% ethanol before embedded in paraffin. Then small intestine in paraffin were sectioned and dewaxed to water. Tissue section performed antigen retrieval with citrate antigen repair buffer (pH 6.0) followed by autofluorescence quenching and serum blocking. Tissues were incubated with mouse IL-22 antibody (Servicebio, Wuhan, China) at 4 °C overnight, and then incubated with CY3-conjugated goat anti-rabbit secondary antibody (Servicebio, Wuhan, China). DAPI (Servicebio, Wuhan, China) was used for nucleus staining. Cells were visualized by fluorescence microscope (Nikon NIKON ECLIPSE C1, Tokyo, Japan).

### 4.12. Statistics Analysis

Statistical analysis was performed using GraphPad Prism 6.0 (San Diego, CA, USA). Statistical comparisons between groups were evaluated by the Student’s unpaired (2-tailed) *t*-tests. Data are presented as the mean ± SEM. *p* value < 0.05 was considered statistically significant and labelled with *, *p* < 0.01 was labeled with **, and *p* < 0.001 was labeled with ***, respectively.

## Figures and Tables

**Table 1:** Primer sequences for over-lap PCR.

Primer Name	Sequence
hIL-23R-SP-sense	5′-CCAAAGCTT^1^GCCACCATGGGTAATCAGG-3′
hIL-23R-SP-antisense	5′-TGGCGGTCCATGACACCAGCTGAAG-3′
hIL-23R-CHR-sense	5′-GGTGTCATGGACCGCCAGATATTCCTG-3′
hIL-23R-CHR-antisense	5′-GACTTGGGCTC^1^ATGAAAAAACGGTGAGCTC-3′
hIgG-Fc-sense	5′-CCGTTTTTTCAT^1^GAGCCCAAGTCCTGCG-3′
hIgG-Fc-antisense	5’-CCTCTCGAG^1^TCACTTGCCGGGGGAC-3′

^1^ “ ” indicates the enzyme site and “ ” denotes the corresponding protective base.

**Table 2:** Quantitative real-time polymerase chain reaction (qPCR) primer sequences.

Primer Name	Sequence
mIL-23R forward	5′-AGAGACACTGATTTGTGGGAAAG-3′
mIL-23R reverse	5′-GTTCCAGGTGCATGTCATGTT-3′
mRORγt forward	5′-ACCTCTTTTCACGGGAGGA-3′
mRORγt reverse	5′-TCCCACATCTC CCACATTG-3′
mIL-17A forward	5′-CAGGGAGAGCTTCATCTGTGT-3′
mIL-17A reverse	5′- GCTGAGCTTTGAGGGATGAT-3′
mCCL20 forward	5′-CGACTGTTGCCTCTCGTACA-3′
mCCL20 reverse	5′-AGGAGGTTCACAGCCCTTTT-3′
mIL-22 forward	5′-TTGTGCGATCTCTGATGGCT-3′
mIL-22 reverse	5′-CCAGCATAAAGGTGCGGTTG-3′
mβ-actin forward	5′-TAAGGCCAACCGTGAAAAG -3′
mβ-actin reverse	5′-ACCAGAGGCATACAGGGACA-3′
